# Health-related quality of life among women and men living with migraine: a Canada-wide cross-sectional study

**DOI:** 10.1186/s10194-024-01882-4

**Published:** 2024-10-09

**Authors:** Alexander C.T. Tam, Hiten Naik, Logan Trenaman, Larry Lynd, Wei Zhang

**Affiliations:** 1 Centre for Advancing Health Outcomes, Providence Research, Vancouver, BC Canada; 2 Department of Medicine, The University of British Columbia, Vancouver, BC Canada; 3 Faculty of Pharmaceutical Sciences, The University of British Columbia, 2405 Wesbrook Mall, V6T 1Z3 Vancouver, BC Canada; 4 Department of Health Systems and Population Health, School of Public Health, University of Washington, Seattle, WA USA

**Keywords:** Health-related quality of life [MeSH], Migraine disorders [MeSH], Patient reported outcome measures [MeSH], Disease Burden [MeSH], Gender-based analysis

## Abstract

**Background:**

Migraine is a prevalent neurologic disorder that affects women more than men. Examining health-related quality of life (HRQoL) by gender can aid decision makers in prioritizing future treatment and prevention programs. We aimed to quantify HRQoL by different levels of migraine disability and by gender.

**Methods:**

As part of a Canada-wide cross-sectional study, we administered an online survey to employed adults who self-reported a diagnosis of migraine. Migraine disability level was assessed using the Migraine Disability Assessment questionnaire (MIDAS). MIDAS scores were used to categorize respondents as having little to no, mild, moderate, or severe level of migraine-related disability. Physical and mental component summary scores (PCS and MCS) and health utilities were derived from responses to the Veterans Rand 12 Item Health Survey. PCS, MCS, and health utilities were summarized by migraine-related disability levels and gender. Covariate-adjusted linear regressions were used to examine the association between migraine disability level and health utility by gender.

**Results:**

A total of 441 participants completed the survey. The sample was predominantly women (60.1%), White race (75.5%), and had a mean age of 37 years. Mean health utility, PCS, and MCS scores were 0.61 (0.22), 45.0 (7.7), and 43.4 (11.0), respectively. All three scores decreased with increased migraine disability level. Gender differences on HRQoL within each migraine disability level were not statistically significant, except in the little to no disability level where women had lower mean MCS scores and health utility relative to men [mean (SD) MCS: women 44.0 (11.3); men 55.1 (8.1), *p* < 0.001; health utility: women 0.66 (0.18); men 0.81 (0.18), *p* < 0.001]. Linear regressions showed women with severe migraine-related disability had reduced health utility compared to women with little to no disability [adjusted difference: -0.16 (95%CI -0.24,-0.09)]. Associations among men increased in magnitude with migraine disability level [adjusted differences: mild − 0.16 (95%CI -0.24,-0.09); moderate − 0.18 (95%CI -0.26,-0.10); severe − 0.28 (95%CI -0.37,-0.20)].

**Conclusions:**

Findings contribute to the literature on the association between migraine disability level and HRQoL by examining trends by gender. Model results emphasize the importance of future treatments reducing severe disability due to migraine among both women and men.

**Supplementary Information:**

The online version contains supplementary material available at 10.1186/s10194-024-01882-4.

## Background

Estimates suggest that 8.3–10.2% of Canadians [1] and approximately 1.1 billion people worldwide are affected by migraine, with prevalence highest during core working ages [2–4]. It is among the top leading causes of disability in people under 50 years of age [3, 5, 6]. Additionally, the humanistic and economic burden of migraine is significant: it is comorbid with other chronic conditions such as rheumatoid arthritis, neurological disorders, depression, anxiety, and stroke [1, 2]; it is associated with reduced work productivity [7–9]; and, it is linked to increased health care resource utilization [1]. Interventions including pharmacological preventative and acute treatments [10, 11], as well as non-pharmacological interventions such as cognitive behavioural therapy, exercise, and relaxation therapy [12–14], can relieve symptoms and improve health-related quality of life (HRQoL).

Sex and gender may be important considerations with respect to migraine prevention and treatment. Migraine disorders are more prevalent in women, and studies have suggested that among those with migraine, attacks may also be more severe in women [2, 15–17]. However, it is less clear whether there are gender differences in HRQoL among people living with migraine. Understanding these differences would help determine whether gender should be considered when evaluating existing and emerging migraine therapies.

Many original studies have examined HRQoL among people with migraine [1, 18], yet there have been comparably fewer studies that estimate health utility values associated with migraine disability levels [19–22]. Health utilities represent patients’ preferences, or values, of different health states or outcomes. These are typically expressed on a scale of 0 to 1, where 0 indicates death and 1 indicates perfect health. In addition to providing a single, overall assessment of a patients’ HRQoL [23], health utilities enable consistent comparisons of different interventions in terms of their improvement in health [24]. This is critical for the conduct of cost-utility analyses of health technologies where improvements are usually represented in quality-adjusted life-years gained. Existing studies have focused on the disutility associated with the painfulness of migraine attacks or the frequency of headaches or different types of migraine [19–22], but there is a lack of evidence on utilities associated with different levels of migraine disability and even less in Canada, and by gender [1, 18].

To this end, we aimed to measure HRQoL for different levels of migraine disability by gender in Canada. Estimates generated from this study can help inform analysts and, subsequently, decision makers by contributing health utilities that may be useful for future economic evaluations of emerging treatment and prevention strategies.

## Methods

### Study design and participants

This was a cross-sectional study of a broader study that examined productivity loss among people with different diseases (migraines, atopic dermatitis, alopecia areata), in which participants completed an online questionnaire administered using Qualtrics (Provo, Utah) [25]. Participants were recruited throughout Canada from an *Ipsos* market research panel. Potential participants were eligible if they were 19 years of age or older, employed, residents of Canada, able to read and understand English or French, and if they self-reported having migraine that was expected to last or have already lasted 6 months, and that was diagnosed by a health professional. We did not collect information related to the form of migraine the participant had, such as chronic migraine or migraine with and without aura. Recruitment and data collection activities for the questionnaire for the migraine population began on December 4, 2023, and concluded on February 12, 2024. We set survey quotas to ensure there was an even distribution of respondents in terms of their migraine disability.

The questionnaire was designed in consultation with one Patient Partner living with migraine and two Patient Partners (one living with atopic dermatitis and one with alopecia areata). Three versions of the questionnaire were prepared, corresponding to the three diseases of interest in the broader study. The questionnaires shared the same socio-demographic questions, productivity loss questions, and quality of life questions (Veterans RAND 12 Item Health Survey, described below). The questionnaires differed on disease-specific questions such as self-reported clinical history, healthcare experience, and on measures of disease severity or disability. Patient Partners for migraine and atopic dermatitis were recruited from REACH BC, a research opportunity platform through which patients can browse and voluntarily sign up for Patient Partner or research study opportunities. The Patient Partner living with alopecia areata was recruited from the Canadian Alopecia Areata Foundation. Drafts of the disease-specific questionnaires were piloted in 3 patients with a history of migraine, 3 patients with atopic dermatitis, and 1 patient with alopecia areata. Patients for pilot testing were recruited from REACH BC. Patients who participated in the pilot provided written feedback, as well as attended a debriefing interview during which the interviewer asked about their experience completing the questionnaire. Written feedback from pilot testing and Patient Partners and interview notes were reviewed by the research team and changes were agreed upon by consensus. Updated versions of the questionnaires were circulated to Patient Partners to review whether changes were aligned with their feedback. The main feedback specific to the migraine questionnaire was to administer it in dark mode.

This study was approved by The University of British Columbia Research Ethics Board (REB# H22-03211). All participants provided consent electronically before initiating the questionnaire. We followed the Strengthening the Reporting of Observational Studies in Epidemiology (STROBE) guidelines for reporting observational studies [26].

### Migraine disability level

Migraine disability level was assessed using the Migraine Disability Assessment (MIDAS) questionnaire [27]. The MIDAS was selected given the availability of cut-offs for classifying respondents into levels of disability and because it requires fewer items to administer compared to other tools such as the Migraine-Specific Quality-of-Life Questionnaire [28, 29].The MIDAS comprises 5 items which ask about the number of days in the past 3 months that were affected by migraine: the number of missed work or school days; missed household chores days; missed non-work activity days; days at work or school where productivity was reduced by half or more; and days in which household work reduced by half or more [27]. The total MIDAS score is the sum of days from each item [27]. We categorized participants as having little to no migraine-related disability (MIDAS score 0–5), mild migraine-related disability (MIDAS score 6–10), moderate migraine-related disability (MIDAS score 11–20), or severe migraine-related disability (MIDAS score ≥ 21) [27]. The MIDAS also includes questions about the number of days in the last 3 months in which the respondent had a headache and the painfulness of headaches on a scale of 0 to 10, with 0 being no pain at all and 10 being pain as bad as it can be. These are not included in the MIDAS disability scoring algorithm, but we included them as separate independent variables in analyses.

### Outcomes

Our primary measure of HRQoL was health utility as derived from the Veterans RAND 12-item (VR-12). The Veterans RAND 12 Item Health Survey was developed from the Veterans RAND 36 Item Health Survey which was developed and modified from the original RAND version of the 36-item Health Survey version 1.0 (also known as the “MOS SF-36”) [30, 31]. The VR-12 is a generic HRQoL instrument that includes fourteen items. The first twelve correspond to eight health domains (physical functioning, social functioning, role limitations due to physical problems, role limitation due to emotional problems, bodily pain, mental health, vitality, and general health) while the remaining two items capture change in physical and emotional health over the past year. We calculated health utility values using an algorithm described by Bansback et al. [32]. The algorithm calculates disutility values subtracted from an initial value of 1.000 (full health). The possible range for health utility derived from this algorithm is − 0.589 to 1.000.

We also calculated the physical component summary (PCS) and mental component summary (MCS) scores based on the item responses [30, 31]. The summary scores were calculated using an algorithm described in Selim et al. [31]. Briefly, the algorithm involves applying regression coefficients (“weights”) to dummy-coded response items. The latest updated weights were calculated by the algorithm developers using multiple regressions predicting PCS and MCS based on data from earlier studies, applying the coefficients to updated data, and correcting for contextual factors and scale [31]. Separate sets of weights are available for responses collected over the phone and through self-administration (i.e., computer or paper surveys). The score is computed as the sum of the weighted items and a constant term, which produces T-scores with a mean of 50 and a standard deviation of 10. The algorithm code and necessary input files were obtained with permission from the developers [31]. We inputted the data from our sample into the algorithm and specified weights for self-administered responses, which then produced the PCS and MCS scores.

### Statistical analysis

Data were summarized according to migraine-related disability levels in the overall sample and separately by women and men. These summaries were means and standard deviations for continuous variables and frequencies and percentages for categorical variables.

We then used ordinary least squares (OLS) regressions to measure the association between migraine-related disability level and health utility while adjusting for potential confounding variables. These additional covariates were age (years, continuous); gender (women/men); ethnicity (White/non-White); marital status (not married or common-law/married or common-law); education attainment (University or college education/ No university or college education); household income (<$50,000/$50,000- $99,999/$100,000- $149,999/≥$150,000); time since migraine diagnosis (years, continuous); and number of comorbidities (0/1/≥2). The selection of OLS to model health utilities was informed by prior research which compared different approaches [35].

We examined gender differences by integrating the general principles outlined in the *Sex- and Gender-Based Analysis* guidance from the Canadian Institutes of Health Research [33]. First, we used gender-stratified regression models in which we did not include the gender variable in the covariate adjustments. We then used separate regression models with interaction terms between gender and migraine-related disability level. Additional analyses included substituting migraine-related disability level with headache painfulness and headache frequency in all models, and using MIDAS scores as a continuous independent variable in the interaction term models.

Statistical tests were two-sided and the threshold for significance was *p* < 0.05. Analyses were performed using R statistical software version 4.3.3 and Stata (15.1, StataCorp LLC, College Station, TX).

## Results

Table 1 presents the characteristics of the analytic sample. A total of 855 potential participants accessed and started the survey, and 455 (response rate: 53.2%) completed the survey (Supplementary Fig. 1). There were more women than men in the overall sample and with mild, moderate, and severe migraine-related disability levels. The mean (SD) age and age at diagnosis were 37.7 years (10.9) and 24.0 years (9.7), respectively. Almost two-thirds of the sample reported one or more comorbidities, with those with a severe migraine-related disability level having the highest proportion reporting two or more comorbidities out of all the migraine-related disability levels. The mean pain of headaches on a scale of 0 to 10, corresponding to each migraine-related disability level, was 4.0 (2.9) for little to no, 5.5 (2.2) for mild, 6.3 (1.8) for moderate, and 7.1 (1.3) for severe. The number of days having a headache also increased with migraine-related disability: 2.9 (6.3), 4.7 (4.5), 8.0 (7.4), and 19.0 (19.3) days, respectively. Women had a higher mean (SD) level of pain and a higher mean number of days of headache than men: 6.4 (2.0) compared to 4.8 (2.6) and 10.2 (14.0) compared to 6.4 (9.9), respectively (Supplementary Table 1). Women also had higher mean MIDAS scores and thus had a higher proportion of severe migraine than men.

**Table 1:** Sociodemographic and clinical characteristics of the analytic sample

Characteristic	Migraine disability level				All *N* (%)
	Little to no *N* (%)	Mild *N* (%)	Moderate *N* (%)	Severe *N* (%)	
**Total**,** row %**	109 (24.7)	111 (25.2)	111 (25.2)	110 (24.9)	441 (100)
**Gender**					
Man	65 (59.6)	42 (37.8)	38 (34.2)	31 (28.2)	176 (39.9)
Woman	44 (40.4)	69 (62.2)	73 (65.8)	79 (71.8)	265 (60.1)
**Age**					
Mean (SD)	36.6 (12.7)	38.8 (10.1)	38.2 (9.5)	37.3 (11.0)	37.7 (10.9)
**Age at migraine diagnosis**					
Mean (SD)	23.4 (9.6)	26.5 (8.9)	23.3 (9.1)	22.8 (11.0)	24.0 (9.7)
**Years with migraine**					
Mean (SD)	13.2 (12.5)	12.2 (11.6)	14.9 (11.1)	14.5 (11.4)	13.7 (11.7)
**Race/ ethnicity**					
Other race/ ethnicity*	23 (23.1)	20 (18.0)	26 (23.4)	39 (35.5)	108 (24.5)
White	86 (78.9)	91 (82.0)	85 (76.6)	71 (64.5)	333 (75.5)
**Marital status**					
Not married or common-law	66 (60.6)	35 (31.5)	38 (34.2)	49 (44.5)	188 (42.6)
Married or common-law	43 (39.4)	76 (68.5)	73 (65.8)	61 (55.5)	253 (57.4)
**Education**					
No university or college education	63 (57.8)	55 (49.5)	66 (59.5)	64 (58.2)	248 (56.2)
University or college education	46 (42.2)	56 (50.5)	45 (40.5)	46 (41.8)	193 (43.8)
**Household income**					
<$50,000	16 (14.7)	16 (14.4)	20 (18.0)	24 (21.8)	76 (17.2)
$50,000- $99,999	17 (15.6)	27 (24.3)	23 (20.7)	37 (33.6)	104 (23.6)
$100,000- $149,999	36 (33.0)	40 (36.0)	35 (31.5)	28 (25.5)	139 (31.5)
≥$150,000	40 (36.7)	28 (25.2)	33 (29.7)	21 (19.1)	122 (27.7)
**Number of comorbidities** ^**a**^					
0	57 (52.3)	36 (32.4)	33 (29.7)	31 (28.2)	157 (35.6)
1	35 (32.1)	47 (42.3)	45 (40.5)	29 (26.4)	156 (35.4)
≥ 2	17 (15.6)	28 (25.2)	33 (29.7)	50 (45.5)	128 (29.0)
**Pain of headache (0 to 10)**					
Mean (SD)	4.0 (2.9)	5.5 (2.2)	6.3 (1.8)	7.1 (1.3)	5.7 (2.4)
**Days in the last 3 mo. having a headache**					
Mean (SD)	2.9 (6.3)	4.7 (4.5)	8.0 (7.4)	19.0 (19.3)	8.7 (12.7)

**Legend**: Migraine disability levels were determined using the Migraine Disability Assessment questionnaire. *Other race/ethnicity includes South Asian (e.g., East Indian, Pakistani, Sri Lankan, etc.), Chinese, First Nations, Southeast Asian (e.g., Vietnamese, Cambodian, Malaysian, Laotian, etc.), West Asian, Filipino, Latin American, Métis, Korean, Japanese, Arab, Inuit, Black, Indigenous/ Aboriginal (not included elsewhere), Other, and mixed (i.e., more than one) ethnicities. ^**a**^Comorbidities include asthma, arthritis or osteoporosis, back problems, cancer, cardiovascular disease, chronic obstructive pulmonary disease (COPD), diabetes, mental health conditions, neurologic conditions, digestive diseases, fibromyalgia or chronic fatigue syndrome, kidney disease, liver disease or gallbladder problems, other. Abbreviations: SD = standard deviation

Table 2 presents the mean component scores and health utility index scores by gender and migraine-related disability level. In the overall sample, the means for all three outcomes decreased as migraine-related disability levels increased. Means for PCS and MCS were numerically similar to each other in each migraine-related disability level; although, there was a more pronounced difference in the severe migraine-related disability level [mean MCS (SD): 35.5 (9.7); PCS: 41.9 (7.9)]. Gender differences were few: among people with little to no migraine-related disability, women had lower mean MCS [women 44.0 (11.3); men 55.1 (8.1), *p* < 0.001] and health utility scores [women0.66 (0.18); men 0.81 (0.18), *p* < 0.001] compared to men whereas PCS scores were comparable across genders. There were no other statistically significant differences in scores across genders in the mild, moderate, and severe levels. Supplementary Table 2 presents the counts of the responses to the VR-12 items. The lower MCS scores among those with a severe migraine-related disability level may be attributed to a marked increase in the proportion of participants reporting feeling calm and peaceful “none of the time” or “a little of the time” (proportion among severe 30.0%, up from 11.7% among moderate disability) and feeling downhearted and blue “all of the time” or “most of the time” (25.5%, up from 10.8% among moderate disability).

**Table 2:** Component scores and health utility index scores based on the VR-12 by gender and by MIDAS disability level

MIDAS		Women (*N* = 265)	Men (*N* = 176)	Total (*N* = 441)	*p* value
**Overall**	**PCS12**				0.046
	Mean (SD)	44.4 (7.9)	45.9 (7.2)	45.0 (7.7)	
	Median (Q1, Q3)	44.6 (39.5, 50.2)	46.0 (40.3, 51.9)	45.1 (39.9, 51.2)	
	**MCS12**				< 0.001
	Mean (SD)	41.1 (10.4)	47.0 (11.1)	43.4 (11.0)	
	Median (Q1, Q3)	41.2 (35.1, 48.1)	48.6 (39.4, 56.1)	44.1 (36.7, 51.8)	
	**VR12 utility index**				< 0.001
	Mean (SD)	0.58 (0.22)	0.67 (0.21)	0.61 (0.22)	
	Median (Q1, Q3)	0.64 (0.46, 0.73)	0.70 (0.55, 0.83)	0.68 (0.51, 0.75)	
**Little to no**	**PCS12**				0.066
	Mean (SD)	49.1 (7.4)	51.4 (5.5)	50.5 (6.4)	
	Median (Q1, Q3)	50.6 (44.7, 54.6)	52.1 (50.7, 55.0)	52.0 (47.9, 54.8)	
	**MCS12**				< 0.001
	Mean (SD)	44.0 (11.3)	55.1 (8.1)	50.6 (10.9)	
	Median (Q1, Q3)	46.0 (37.7, 52.1)	58.2 (52.6, 59.9)	53.2 (46.1, 59.1)	
	**VR12 utility index**				< 0.001
	Mean (SD)	0.66 (0.18)	0.81 (0.18)	0.75 (0.19)	
	Median (Q1, Q3)	0.70 (0.60, 0.78)	0.85 (0.73, 0.91)	0.79 (0.68, 0.86)	
**Mild**	**PCS12**				0.083
	Mean (SD)	44.8 (5.5)	42.8 (6.0)	44.0 (5.8)	
	Median (Q1, Q3)	44.5 (40.7, 49.0)	42.1 (37.9, 47.2)	43.3 (40.3, 48.6)	
	**MCS12**				0.682
	Mean (SD)	44.8 (9.2)	45.5 (8.6)	45.1 (8.9)	
	Median (Q1, Q3)	44.6 (39.7, 52.3)	46.0 (40.3, 51.6)	45.0 (39.9, 51.8)	
	**VR12 utility index**				0.952
	Mean (SD)	0.63 (0.16)	0.64 (0.15)	0.63 (0.15)	
	Median (Q1, Q3)	0.68 (0.53, 0.73)	0.68 (0.56, 0.72)	0.68 (0.54, 0.73)	
**Moderate**	**PCS12**				0.331
	Mean (SD)	43.9 (8.1)	42.4 (6.5)	43.4 (7.6)	
	Median (Q1, Q3)	44.0 (39.0, 50.2)	41.3 (38.4, 46.2)	43.3 (38.8, 48.6)	
	**MCS12**				0.319
	Mean (SD)	42.1 (9.3)	43.9 (8.5)	42.7 (9.0)	
	Median (Q1, Q3)	42.9 (35.3, 48.1)	44.1 (38.7, 51.0)	43.2 (36.3, 48.5)	
	**VR12 utility index**				0.747
	Mean (SD)	0.60 (0.18)	0.61 (0.14)	0.60 (0.16)	
	Median (Q1, Q3)	0.65 (0.51, 0.73)	0.64 (0.53, 0.71)	0.64 (0.51, 0.72)	
**Severe**	**PCS12**				0.679
	Mean (SD)	41.8 (8.6)	42.4 (5.5)	41.9 (7.9)	
	Median (Q1, Q3)	42.2 (37.1, 47.7)	42.4 (38.2, 47.1)	42.3 (38.0, 47.4)	
	**MCS12**				0.854
	Mean (SD)	35.4 (9.5)	35.7 (10.1)	35.5 (9.7)	
	Median (Q1, Q3)	36.9 (29.6, 41.5)	37.2 (29.4, 40.9)	37.1 (29.5, 41.2)	
	**VR12 utility index**				0.761
	Mean (SD)	0.46 (0.27)	0.48 (0.24)	0.47 (0.26)	
	Median (Q1, Q3)	0.51 (0.36, 0.68)	0.55 (0.36, 0.66)	0.53 (0.36, 0.68)	

**Legend**: *p* values are based on one-way ANOVA comparing women and men. Abbreviations: MIDAS = Migraine Disability Assessment; MCS12 = Mental Component Score; PCS12 = Physical Component Socre; Q1 = 1st quartile; Q3 = 3rd quartile; SD = standard deviation; VR-12 = Veterans Rand 12-item Health Survey

**Fig. 1:**
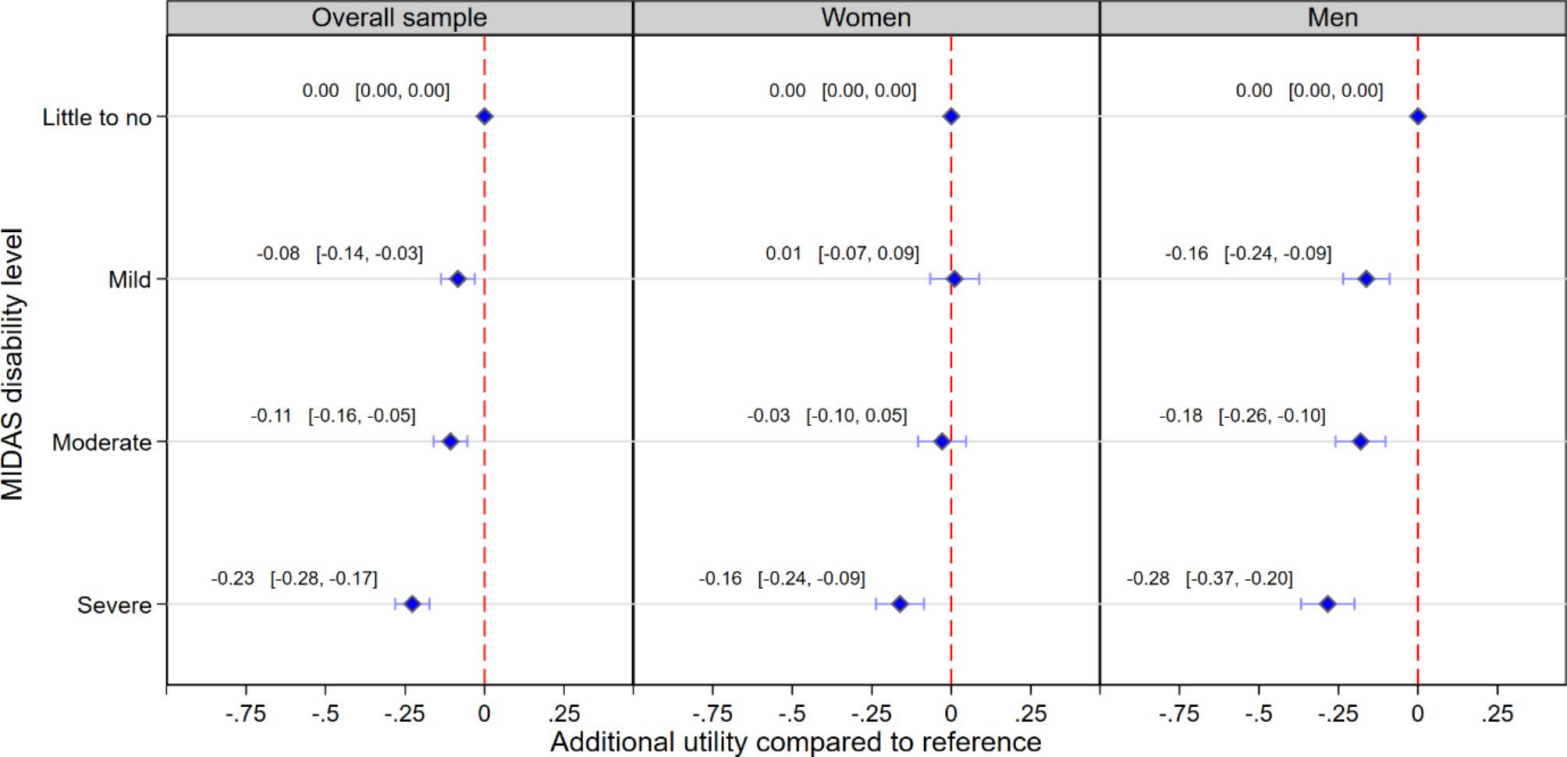
Multiple regression models for the association between MIDAS disability level and health utility index score derived from the VR-12. **Legend**: Abbreviations: MIDAS = Migraine Disability Assessment; VR-12 = Veterans Rand 12-item Health Survey. Models were adjusted for age, White ethnicity, education attainment, marital status, household income, migraine duration (since diagnosis), and the number of comorbidities. Models including the overall sample also adjusted for gender

Adjusted regression models showed mild, moderate, and severe migraine-related disability levels were each associated with decreased health utility compared to little to no disability [difference: -0.08 (95% Confidence Interval (CI) : -0.14,-0.03); difference: -0.11 (95%CI: -0.16,-0.05); difference: -0.23 (95%CI: -0.28,-0.17), respectively] (Fig. 1 and Supplementary Table 3). Increasing ratings of headache painfulness and increasing headache frequency in the last 3 months were also associated with decreased health utility [change per 1-point increase: -0.03 (95%CI: -0.04,-0.02); change per 1-day increase: -0.0052 (95%CI: -0.0067,-0.0037), respectively] (Table 3). Gender-stratified regression models revealed different patterns in the associations between migraine-related disability level and health utility. Among women, the associations for mild and moderate migraine-related disability levels were numerically small and non-statistically significant [difference: 0.01 (95%CI: -0.07,0.09); difference: -0.03 (95%CI: -0.10,0.05), respectively]. Whereas a severe migraine-related disability level was associated with a decrease of 0.16 (95%CI: -0.24,-0.09) in health utility compared to women with little to no migraine-related disability. By contrast, all three migraine-related disability levels were statistically significantly associated with decreased health utility compared to reference among men. The associations followed a similar gradient as in the overall sample, increasing in magnitude from mild [difference: -0.16 (95%CI: -0.24,-0.09)] to severe [difference: -0.28 (95%CI: -0.37,-0.20)]. Associations for headache pain and headache frequency were similar in magnitude among both women and men. All coefficients from each set of adjusted regression models are reported in Supplementary Tables 3–5.

**Table 3:** Multiple regression models for the association between headache pain and headache frequency with health utility index score derived from the VR-12

Independent variable	Overall Sample Coefficient (95%CI)		Women Coefficient (95%CI)		Men Coefficient (95%CI)
**Headache pain (0 to 10)**	-0.03 [-0.04,-0.02]***		-0.02 [-0.03,-0.01]**		-0.03 [-0.05,-0.02]***
**Headache frequency (days in last 3 mo.)**	-0.0052 [-0.0067,-0.0037]***		-0.0048 [-0.0065,-0.0030]***		-0.0063 [-0.0094,-0.0032]***

**Legend**: Abbreviations: VR-12 = Veterans Rand 12-item Health Survey. Models were adjusted for age, White ethnicity, education attainment, marital status, household income, migraine duration (since diagnosis), and the number of comorbidities. Models including the overall sample also adjusted for gender. *p < 0.05; **p < 0.01; ***p < 0.001

Adjusted regression models using interaction terms rather than gender-stratification showed that the association between migraine-related disability level and health utility did not statistically significantly differ across women and men. At the reference level, which was changed to severe migraine disability level, women had comparable health utility to men [difference: -0.02 (95%CI: -0.10,0.06)] (Supplementary Table 6). Interaction terms were similarly non-statistically significant. When MIDAS scores were used instead of MIDAS migraine-related disability level, the gender coefficient showed that women had lower health utility compared to men when MIDAS scores were equal to 0 [difference: -0.06 (95%CI: -0.11,-0.01)]; however, the interaction term was non-statistically significant (Supplementary Table 7). Similar to the gender-stratified regression models, interaction-term models showed the associations for headache pain and headache frequency were similar among women and men (Supplementary Table 7).

## Discussion

In this cross-sectional study, we examined the associations between migraine-related disability and HRQoL and explored gender differences. In bivariate comparisons by gender, we found women had lower mean MCS scores and health utility in the little to no migraine disability group compared to men; and we found no other statistically significant differences across genders among the other disability levels. In covariate-adjusted regressions in the whole sample, we found that increased migraine-related disability was associated with decreased health utility compared to little to no disability. Gender-stratified analyses showed different patterns in the associations across genders. Among women, severe migraine-related disability was associated with lower health utility, whereas mild and moderate migraine-related disability levels showed similar health utility compared to little to no disability. Among men, the magnitude of the associations increased with each disability level compared to little to no disability. Analyses using gender interaction terms showed that the association between migraine-related disability and health utility did not differ statistically across women and men.

To aid in the interpretation of the HRQoL values, we can compare our estimates to the Canadian population norms as reported in Trenaman et al. [34]. Compared to the gender-specific population norms for Canadians, women and men with little to no migraine disability had comparable mean PCS scores (i.e., about 1 point of difference), but nominally different MCS scores (about 5 points of difference). However, those with mild, moderate, or severe disability levels had consistently lower component scores. Notably, those with severe disability had mean MCS scores that were > 10 points lower than the norm. Similarly, there was a stark difference between the mean health utilities among people with severe migraine disability and the population norms.

Other studies have examined migraine HRQoL (see the reviews from Graves et al. [1] and Abu Bakar et al. [18]), but comparably fewer studies have examined the relationship between HRQoL and indicators of disability or disease severity [18–20, 35, 36]. For example, Raggi et al. [35] examined domain scores for the 36-Item Short Form Health Survey (SF-36) and MIDAS disability levels among patients attending a specialized medical clinic. As found in the present study with the VR-12, mean PCS and MCS scores calculated from the SF-36 increased with higher disability levels, and mean MCS scores were nominally lower than mean PCS scores [35]. Similarly, in a population-based study in the UK, Lipton et al. [36] compared SF-36 scores across categories of disease severity defined based on the number of work days in which respondents were affected by a headache. Those with migraine had statistically significantly lower PCS and MCS relative to healthy controls.

Studies that have estimated health state utility values according to migraine disability or severity also corroborate results from the present study despite differences in the measure of HRQoL or migraine-related disability [19–22]. Domitriz and Golicki [19] compared EuroQol-5 Dimension-5 Level (EQ-5D-5 L) across those with episodic and chronic migraine. They found that both chronic and episodic migraine were associated with reduced HRQoL compared to matched controls, and that the associations were larger (i.e., greater reductions) for those with chronic migraines. In a multi-European country study, Doane et al. [14] found that the number of headache-free days in the past 30 days was significantly associated with higher utility scores on EQ-5D-5 L and on SF Six-Dimension. Our measure of headache frequency had a longer recall period of 3 months, but our estimated coefficient indicates the same direction and a similar magnitude of the association. Stafford et al. [21] examined EQ-5D-3 Level (EQ-5D-3 L) utility values by self-reported severity of participants’ most recent migraine attack in the last 7 days among patients recruited from UK migraine support groups. Using data from a US clinical trial evaluating the efficacy of telcagepant, Xu et al. [22] estimated health disutility associated with migraine attacks by the degree of baseline headache pain. The estimated disutilities in both Stafford et al. [21] and Xu et al. [22] were in range of each other, but comparisons to the current study could not be made as MIDAS does not have established cut-offs to create groups of pain intensity and because it asks respondents to consider average pain over a recall period of 3 months.

Overall, our findings are aligned in the same direction as the literature. However, our major contribution was additionally analyzing health utilities for women and men separately. In one other Canada-wide cross-sectional study, unadjusted estimates for health utility as measured using the Health Utilities Index were lower among males than females [37]. However, males were also more likely than females to report poor or fair health and were less likely to report headache-related light sensitivity and headache-related limitations in their ability to work. Thus, gender differences in health utility may be confounded by other comorbid health conditions or disease severity – a limitation fully acknowledged by the authors [37]. By contrast, our unadjusted estimates indicated that women with little to no migraine-related disability had lower health utility and MCS compared to men. HRQoL outcomes were more comparable within each of the mild, moderate, and severe disability levels across genders. However, our adjusted estimates suggested gendered patterning. Of note, women with little to no migraine-related disability had similar health utility as women with mild and moderate disability levels. This warrants attention as this was likely attributed to women with little to no migraine-related disability level having relatively low crude health utility and MCS as shown in the bivariate associations. This implies that even less severe migraine-related disability levels may negatively affect HRQoL among women. It is also worth noting that migraine-related disability as defined using MIDAS does not account for important differences such as women experiencing longer duration of migraine attacks and prolonged recovery periods relative to men [38–40]. In turn, these additional characteristics of migraine negatively impact HRQoL [41]. So while treatments aimed at curtailing severe migraine-related disability are valuable for both women and men, attention should also be given to treatments for women with little to no migraine-related disability to improve HRQoL. Among men, the gradient in the associations also warrant attention. One explanation for the larger decreases in health utility with greater migraine-related disability could be related to accessing treatment [15]. Men are more likely to take no medications or over-the-counter medications and women are more likely to take prescription medication [15]. Relatedly, men are also less likely to contact a headache centre or seek consultations related to their treatment [38, 42], which potentially leads to sub-optimal management of migraine among men.

The present findings should be interpreted in light of the study limitations. Firstly, as a survey-based study, we relied on respondents self-reporting a diagnosis of migraine for inclusion into the study and these were not verified clinically or with headache diaries. Related to this, we also did not collect information to differentiate the types of migraine respondents were diagnosed with – some of which affect only women and people who menstruate (i.e., menstrual migraine) [17]. Further, we did not obtain information about specific medications or frequency of medication use, so we could not adjust for these factors in our model or ascertain the extent of medication overuse in the sample. Finally, as this study was a part of a larger study that primarily focused on productivity, we excluded potential respondents if they reported not working. We also used survey quotas based on age, sex, and migraine disability to ensure a balance across groups. Together, this meant the study sample was not representative of the Canadian population of people living with migraine and this limits the generalizability of results. Despite this, we found that those in the little to no disability group had PCS and MCS scores that were somewhat similar to general population norms, which span a diverse range of socio-demographic characteristics.

## Conclusions

We examined the association between migraine disability and HRQoL, and found that both the physical and mental components of HRQoL, as well as health utility, worsened with increased disability in our unadjusted analysis. Gender-specific patterns emerged in covariate-adjusted analysis. Findings indicated that severe migraine-related disability was associated with worse health utility compared to little to no disability among women and men. However, men with moderate and mild disability also had worse health utility, whereas women with these levels of disability had comparable health utilities to reference. These findings point to the importance of identifying treatment and management strategies that can improve HRQoL among those with the most severe migraine-related disability, and the importance of not overlooking mild migraine-related disability, especially in men. The finding that women with little to no disability have similar HRQoL compared to women with mild and moderate migraine disability warrant further research. The findings from this study are directly relevant for future economic modeling to assess the cost-effectiveness of interventions. Given the patterning we found, models should include gender- and disability level-specific estimates of HRQoL. Furthermore, clinicians treating those with migraine should also not overlook milder disabilities, as HRQoL burden may still be high. Overall, this study underscores the importance of taking a gendered approach to migraine research and treatment as the associations between disability and HRQoL are nuanced.

## Electronic supplementary material

Below is the link to the electronic supplementary material.

Supplementary Material 1

## Data Availability

The datasets used and/or analyzed during the current study are not publicly available but are available from the corresponding author upon reasonable request.

## Abbreviations

CI: Confidence interval
EQ-5D-3L: EuroQol-5 Dimension-3 Level
EQ-5D-5L: EuroQol-5 Dimension-5 Level
HRQoL: Health-related quality of life
MCS: Mental Component Summary
MIDAS: Migraine Disability Assessment
OLS: Ordinary least squares
PCS: Physical Component Summary
SD: Standard deviation
SF-12: 12-Item Short-Form Health Survey
SF-36: 36-Item Short-Form Health Survey
VR-12: Veterans Rand 12 Item Health Survey
