# Supplementary material for: Health-related quality of life among women and men living with migraine: a Canada-wide cross-sectional study
Source: J Headache Pain. 2024 Oct 9;25(1):170. doi: 10.1186/s10194-024-01882-4 (PMC11462961; doi:10.1186/s10194-024-01882-4)
Supplement: Supplementary file 1 — Supplementary Material 1 [file 10194_2024_1882_MOESM1_ESM.docx]

**Supplementary Figure 1**. Recruitment, completion, and selection of study sample.

**Supplementary Table 1**. Gender differences in MIDAS scores, disability level, painfulness and frequency of headaches.

|  | **Women (N=265)** | **Men (N=176)** | **Total (N=441)** |
| --- | --- | --- | --- |
| **MIDAS disability level** |  |  |  |
| Mean (SD) | 22.6 (33.3) | 13.5 (18.0) | 19.0 (28.6) |
| **MIDAS disability level** |  |  |  |
| Little to no n (%) | 44 (16.6%) | 65 (36.9%) | 109 (24.7%) |
| Mild n (%) | 69 (26.0%) | 42 (23.9%) | 111 (25.2%) |
| Moderate n (%) | 73 (27.5%) | 38 (21.6%) | 111 (25.2%) |
| Severe n (%) | 79 (29.8%) | 31 (17.6%) | 110 (24.9%) |
| **MIDAS pain of headache (0 to 10)** |  |  |  |
| Mean (SD) | 6.4 (2.0) | 4.8 (2.6) | 5.7 (2.4) |
| **MIDAS days in the last 3 mo. having a headache** |  |  |  |
| Mean (SD) | 10.2 (14.0) | 6.4 (9.9) | 8.7 (12.7) |

**Legend**: Abbreviations: MIDAS = Migraine Disability Assessment

**Supplementary Table 2**. Responses to the VR-12 by gender and by MIDAS disability level.

| MIDAS |  | Men  (N=176) | Women  (N=265) | Total  (N=441) |
| --- | --- | --- | --- | --- |
| Little to no | General Health (GH1) |  |  |  |
|  | Excellent | 25 (38.5%) | 6 (13.6%) | 31 (28.4%) |
|  | Very good | 24 (36.9%) | 15 (34.1%) | 39 (35.8%) |
|  | Good | 13 (20.0%) | 17 (38.6%) | 30 (27.5%) |
|  | Fair | 3 (4.6%) | 6 (13.6%) | 9 (8.3%) |
|  | Poor | 0 (0.0%) | 0 (0.0%) | 0 (0.0%) |
|  | Limited in Moderate Activities (PF02) |  |  |  |
|  | Yes, limited a lot | 2 (3.1%) | 3 (6.8%) | 5 (4.6%) |
|  | Yes, limited a little | 7 (10.8%) | 10 (22.7%) | 17 (15.6%) |
|  | No, not limited at all | 56 (86.2%) | 31 (70.5%) | 87 (79.8%) |
|  | Limited in Climbing Stairs (PF04) |  |  |  |
|  | Yes, limited a lot | 1 (1.5%) | 3 (6.8%) | 4 (3.7%) |
|  | Yes, limited a little | 9 (13.8%) | 14 (31.8%) | 23 (21.1%) |
|  | No, not limited at all | 55 (84.6%) | 27 (61.4%) | 82 (75.2%) |
|  | Accomplished less as a result of Physical Health (VRP2) |  |  |  |
|  | No, none of the time | 52 (80.0%) | 18 (40.9%) | 70 (64.2%) |
|  | Yes, a little of the time | 6 (9.2%) | 19 (43.2%) | 25 (22.9%) |
|  | Yes, some of the time | 6 (9.2%) | 5 (11.4%) | 11 (10.1%) |
|  | Yes, most of the time | 0 (0.0%) | 2 (4.5%) | 2 (1.8%) |
|  | Yes, all of the time | 1 (1.5%) | 0 (0.0%) | 1 (0.9%) |
|  | Limited in the kind of work or other activities as a result of Physical Health (VRP3) |  |  |  |
|  | No, none of the time | 50 (76.9%) | 22 (50.0%) | 72 (66.1%) |
|  | Yes, a little of the time | 12 (18.5%) | 16 (36.4%) | 28 (25.7%) |
|  | Yes, some of the time | 2 (3.1%) | 4 (9.1%) | 6 (5.5%) |
|  | Yes, most of the time | 0 (0.0%) | 2 (4.5%) | 2 (1.8%) |
|  | Yes, all of the time | 1 (1.5%) | 0 (0.0%) | 1 (0.9%) |
|  | Accomplished less as a result of any Emotional Problems (VRE2) |  |  |  |
|  | No, none of the time | 45 (69.2%) | 16 (36.4%) | 61 (56.0%) |
|  | Yes, a little of the time | 17 (26.2%) | 17 (38.6%) | 34 (31.2%) |
|  | Yes, some of the time | 1 (1.5%) | 7 (15.9%) | 8 (7.3%) |
|  | Yes, most of the time | 1 (1.5%) | 3 (6.8%) | 4 (3.7%) |
|  | Yes, all of the time | 1 (1.5%) | 1 (2.3%) | 2 (1.8%) |
|  | Limited in the kind of work or other activities as a result of any Emotional Problems (VRE3) |  |  |  |
|  | No, none of the time | 48 (73.8%) | 19 (43.2%) | 67 (61.5%) |
|  | Yes, a little of the time | 13 (20.0%) | 15 (34.1%) | 28 (25.7%) |
|  | Yes, some of the time | 3 (4.6%) | 6 (13.6%) | 9 (8.3%) |
|  | Yes, most of the time | 0 (0.0%) | 2 (4.5%) | 2 (1.8%) |
|  | Yes, all of the time | 1 (1.5%) | 2 (4.5%) | 3 (2.8%) |
|  | Pain interfering with normal work (BP2) |  |  |  |
|  | Not at all | 24 (36.9%) | 13 (29.5%) | 37 (33.9%) |
|  | A little bit | 37 (56.9%) | 20 (45.5%) | 57 (52.3%) |
|  | Moderately | 3 (4.6%) | 10 (22.7%) | 13 (11.9%) |
|  | Quite a bit | 0 (0.0%) | 1 (2.3%) | 1 (0.9%) |
|  | Extremely | 1 (1.5%) | 0 (0.0%) | 1 (0.9%) |
|  | Calm and peaceful (MH3) |  |  |  |
|  | All of the time | 26 (40.0%) | 1 (2.3%) | 27 (24.8%) |
|  | Most of the time | 34 (52.3%) | 16 (36.4%) | 50 (45.9%) |
|  | A good bit of the time | 3 (4.6%) | 7 (15.9%) | 10 (9.2%) |
|  | Some of the time | 2 (3.1%) | 13 (29.5%) | 15 (13.8%) |
|  | A little of the time | 0 (0.0%) | 5 (11.4%) | 5 (4.6%) |
|  | None of the time | 0 (0.0%) | 2 (4.5%) | 2 (1.8%) |
|  | A lot of energy (VT2) |  |  |  |
|  | All of the time | 23 (35.4%) | 1 (2.3%) | 24 (22.0%) |
|  | Most of the time | 28 (43.1%) | 14 (31.8%) | 42 (38.5%) |
|  | A good bit of the time | 9 (13.8%) | 11 (25.0%) | 20 (18.3%) |
|  | Some of the time | 5 (7.7%) | 9 (20.5%) | 14 (12.8%) |
|  | A little of the time | 0 (0.0%) | 8 (18.2%) | 8 (7.3%) |
|  | None of the time | 0 (0.0%) | 1 (2.3%) | 1 (0.9%) |
|  | Downhearted and blue (MH4) |  |  |  |
|  | All of the time | 2 (3.1%) | 2 (4.5%) | 4 (3.7%) |
|  | Most of the time | 3 (4.6%) | 3 (6.8%) | 6 (5.5%) |
|  | A good bit of the time | 0 (0.0%) | 8 (18.2%) | 8 (7.3%) |
|  | Some of the time | 3 (4.6%) | 10 (22.7%) | 13 (11.9%) |
|  | A little of the time | 30 (46.2%) | 14 (31.8%) | 44 (40.4%) |
|  | None of the time | 27 (41.5%) | 7 (15.9%) | 34 (31.2%) |
|  | Physical health or emotional problems interfering with social activities (SF2) |  |  |  |
|  | All of the time | 2 (3.1%) | 1 (2.3%) | 3 (2.8%) |
|  | Most of the time | 1 (1.5%) | 5 (11.4%) | 6 (5.5%) |
|  | Some of the time | 2 (3.1%) | 8 (18.2%) | 10 (9.2%) |
|  | A little of the time | 27 (41.5%) | 17 (38.6%) | 44 (40.4%) |
|  | None of the time | 33 (50.8%) | 13 (29.5%) | 46 (42.2%) |
| Mild | General Health (GH1) |  |  |  |
|  | Excellent | 4 (9.5%) | 1 (1.4%) | 5 (4.5%) |
|  | Very good | 14 (33.3%) | 21 (30.4%) | 35 (31.5%) |
|  | Good | 18 (42.9%) | 38 (55.1%) | 56 (50.5%) |
|  | Fair | 6 (14.3%) | 8 (11.6%) | 14 (12.6%) |
|  | Poor | 0 (0.0%) | 1 (1.4%) | 1 (0.9%) |
|  | Limited in Moderate Activities (PF02) |  |  |  |
|  | Yes, limited a lot | 5 (11.9%) | 7 (10.1%) | 12 (10.8%) |
|  | Yes, limited a little | 29 (69.0%) | 28 (40.6%) | 57 (51.4%) |
|  | No, not limited at all | 8 (19.0%) | 34 (49.3%) | 42 (37.8%) |
|  | Limited in Climbing Stairs (PF04) |  |  |  |
|  | Yes, limited a lot | 4 (9.5%) | 5 (7.2%) | 9 (8.1%) |
|  | Yes, limited a little | 25 (59.5%) | 34 (49.3%) | 59 (53.2%) |
|  | No, not limited at all | 13 (31.0%) | 30 (43.5%) | 43 (38.7%) |
|  | Accomplished less as a result of Physical Health (VRP2) |  |  |  |
|  | No, none of the time | 5 (11.9%) | 13 (18.8%) | 18 (16.2%) |
|  | Yes, a little of the time | 12 (28.6%) | 32 (46.4%) | 44 (39.6%) |
|  | Yes, some of the time | 22 (52.4%) | 18 (26.1%) | 40 (36.0%) |
|  | Yes, most of the time | 3 (7.1%) | 5 (7.2%) | 8 (7.2%) |
|  | Yes, all of the time | 0 (0.0%) | 1 (1.4%) | 1 (0.9%) |
|  | Limited in the kind of work or other activities as a result of Physical Health (VRP3) |  |  |  |
|  | No, none of the time | 5 (11.9%) | 24 (34.8%) | 29 (26.1%) |
|  | Yes, a little of the time | 21 (50.0%) | 26 (37.7%) | 47 (42.3%) |
|  | Yes, some of the time | 12 (28.6%) | 14 (20.3%) | 26 (23.4%) |
|  | Yes, most of the time | 4 (9.5%) | 5 (7.2%) | 9 (8.1%) |
|  | Yes, all of the time | 0 (0.0%) | 0 (0.0%) | 0 (0.0%) |
|  | Accomplished less as a result of any Emotional Problems (VRE2) |  |  |  |
|  | No, none of the time | 5 (11.9%) | 21 (30.4%) | 26 (23.4%) |
|  | Yes, a little of the time | 14 (33.3%) | 27 (39.1%) | 41 (36.9%) |
|  | Yes, some of the time | 19 (45.2%) | 14 (20.3%) | 33 (29.7%) |
|  | Yes, most of the time | 4 (9.5%) | 6 (8.7%) | 10 (9.0%) |
|  | Yes, all of the time | 0 (0.0%) | 1 (1.4%) | 1 (0.9%) |
|  | Limited in the kind of work or other activities as a result of any Emotional Problems (VRE3) |  |  |  |
|  | No, none of the time | 10 (23.8%) | 24 (34.8%) | 34 (30.6%) |
|  | Yes, a little of the time | 16 (38.1%) | 23 (33.3%) | 39 (35.1%) |
|  | Yes, some of the time | 13 (31.0%) | 16 (23.2%) | 29 (26.1%) |
|  | Yes, most of the time | 2 (4.8%) | 4 (5.8%) | 6 (5.4%) |
|  | Yes, all of the time | 1 (2.4%) | 2 (2.9%) | 3 (2.7%) |
|  | Pain interfering with normal work (BP2) |  |  |  |
|  | Not at all | 7 (16.7%) | 7 (10.1%) | 14 (12.6%) |
|  | A little bit | 17 (40.5%) | 36 (52.2%) | 53 (47.7%) |
|  | Moderately | 15 (35.7%) | 24 (34.8%) | 39 (35.1%) |
|  | Quite a bit | 3 (7.1%) | 1 (1.4%) | 4 (3.6%) |
|  | Extremely | 0 (0.0%) | 1 (1.4%) | 1 (0.9%) |
|  | Calm and peaceful (MH3) |  |  |  |
|  | All of the time | 3 (7.1%) | 3 (4.3%) | 6 (5.4%) |
|  | Most of the time | 21 (50.0%) | 20 (29.0%) | 41 (36.9%) |
|  | A good bit of the time | 7 (16.7%) | 23 (33.3%) | 30 (27.0%) |
|  | Some of the time | 8 (19.0%) | 17 (24.6%) | 25 (22.5%) |
|  | A little of the time | 3 (7.1%) | 6 (8.7%) | 9 (8.1%) |
|  | None of the time | 0 (0.0%) | 0 (0.0%) | 0 (0.0%) |
|  | A lot of energy (VT2) |  |  |  |
|  | All of the time | 6 (14.3%) | 4 (5.8%) | 10 (9.0%) |
|  | Most of the time | 13 (31.0%) | 24 (34.8%) | 37 (33.3%) |
|  | A good bit of the time | 14 (33.3%) | 18 (26.1%) | 32 (28.8%) |
|  | Some of the time | 6 (14.3%) | 10 (14.5%) | 16 (14.4%) |
|  | A little of the time | 3 (7.1%) | 11 (15.9%) | 14 (12.6%) |
|  | None of the time | 0 (0.0%) | 2 (2.9%) | 2 (1.8%) |
|  | Downhearted and blue (MH4) |  |  |  |
|  | All of the time | 0 (0.0%) | 0 (0.0%) | 0 (0.0%) |
|  | Most of the time | 3 (7.1%) | 8 (11.6%) | 11 (9.9%) |
|  | A good bit of the time | 6 (14.3%) | 9 (13.0%) | 15 (13.5%) |
|  | Some of the time | 13 (31.0%) | 21 (30.4%) | 34 (30.6%) |
|  | A little of the time | 16 (38.1%) | 23 (33.3%) | 39 (35.1%) |
|  | None of the time | 4 (9.5%) | 8 (11.6%) | 12 (10.8%) |
|  | Physical health or emotional problems interfering with social activities (SF2) |  |  |  |
|  | All of the time | 2 (4.8%) | 2 (2.9%) | 4 (3.6%) |
|  | Most of the time | 7 (16.7%) | 11 (15.9%) | 18 (16.2%) |
|  | Some of the time | 13 (31.0%) | 17 (24.6%) | 30 (27.0%) |
|  | A little of the time | 13 (31.0%) | 25 (36.2%) | 38 (34.2%) |
|  | None of the time | 7 (16.7%) | 14 (20.3%) | 21 (18.9%) |
| Moderate | General Health (GH1) |  |  |  |
|  | Excellent | 2 (5.3%) | 7 (9.6%) | 9 (8.1%) |
|  | Very good | 11 (28.9%) | 24 (32.9%) | 35 (31.5%) |
|  | Good | 18 (47.4%) | 32 (43.8%) | 50 (45.0%) |
|  | Fair | 7 (18.4%) | 9 (12.3%) | 16 (14.4%) |
|  | Poor | 0 (0.0%) | 1 (1.4%) | 1 (0.9%) |
|  | Limited in Moderate Activities (PF02) |  |  |  |
|  | Yes, limited a lot | 4 (10.5%) | 9 (12.3%) | 13 (11.7%) |
|  | Yes, limited a little | 18 (47.4%) | 26 (35.6%) | 44 (39.6%) |
|  | No, not limited at all | 16 (42.1%) | 38 (52.1%) | 54 (48.6%) |
|  | Limited in Climbing Stairs (PF04) |  |  |  |
|  | Yes, limited a lot | 4 (10.5%) | 7 (9.6%) | 11 (9.9%) |
|  | Yes, limited a little | 22 (57.9%) | 38 (52.1%) | 60 (54.1%) |
|  | No, not limited at all | 12 (31.6%) | 28 (38.4%) | 40 (36.0%) |
|  | Accomplished less as a result of Physical Health (VRP2) |  |  |  |
|  | No, none of the time | 4 (10.5%) | 14 (19.2%) | 18 (16.2%) |
|  | Yes, a little of the time | 13 (34.2%) | 23 (31.5%) | 36 (32.4%) |
|  | Yes, some of the time | 18 (47.4%) | 27 (37.0%) | 45 (40.5%) |
|  | Yes, most of the time | 3 (7.9%) | 8 (11.0%) | 11 (9.9%) |
|  | Yes, all of the time | 0 (0.0%) | 1 (1.4%) | 1 (0.9%) |
|  | Limited in the kind of work or other activities as a result of Physical Health (VRP3) |  |  |  |
|  | No, none of the time | 6 (15.8%) | 18 (24.7%) | 24 (21.6%) |
|  | Yes, a little of the time | 18 (47.4%) | 30 (41.1%) | 48 (43.2%) |
|  | Yes, some of the time | 12 (31.6%) | 23 (31.5%) | 35 (31.5%) |
|  | Yes, most of the time | 2 (5.3%) | 1 (1.4%) | 3 (2.7%) |
|  | Yes, all of the time | 0 (0.0%) | 1 (1.4%) | 1 (0.9%) |
|  | Accomplished less as a result of any Emotional Problems (VRE2) |  |  |  |
|  | No, none of the time | 6 (15.8%) | 11 (15.1%) | 17 (15.3%) |
|  | Yes, a little of the time | 21 (55.3%) | 32 (43.8%) | 53 (47.7%) |
|  | Yes, some of the time | 9 (23.7%) | 24 (32.9%) | 33 (29.7%) |
|  | Yes, most of the time | 2 (5.3%) | 6 (8.2%) | 8 (7.2%) |
|  | Yes, all of the time | 0 (0.0%) | 0 (0.0%) | 0 (0.0%) |
|  | Limited in the kind of work or other activities as a result of any Emotional Problems (VRE3) |  |  |  |
|  | No, none of the time | 5 (13.2%) | 19 (26.0%) | 24 (21.6%) |
|  | Yes, a little of the time | 14 (36.8%) | 25 (34.2%) | 39 (35.1%) |
|  | Yes, some of the time | 12 (31.6%) | 23 (31.5%) | 35 (31.5%) |
|  | Yes, most of the time | 7 (18.4%) | 5 (6.8%) | 12 (10.8%) |
|  | Yes, all of the time | 0 (0.0%) | 1 (1.4%) | 1 (0.9%) |
|  | Pain interfering with normal work (BP2) |  |  |  |
|  | Not at all | 2 (5.3%) | 5 (6.8%) | 7 (6.3%) |
|  | A little bit | 12 (31.6%) | 31 (42.5%) | 43 (38.7%) |
|  | Moderately | 22 (57.9%) | 29 (39.7%) | 51 (45.9%) |
|  | Quite a bit | 2 (5.3%) | 7 (9.6%) | 9 (8.1%) |
|  | Extremely | 0 (0.0%) | 1 (1.4%) | 1 (0.9%) |
|  | Calm and peaceful (MH3) |  |  |  |
|  | All of the time | 3 (7.9%) | 3 (4.1%) | 6 (5.4%) |
|  | Most of the time | 11 (28.9%) | 21 (28.8%) | 32 (28.8%) |
|  | A good bit of the time | 11 (28.9%) | 17 (23.3%) | 28 (25.2%) |
|  | Some of the time | 11 (28.9%) | 21 (28.8%) | 32 (28.8%) |
|  | A little of the time | 2 (5.3%) | 11 (15.1%) | 13 (11.7%) |
|  | None of the time | 0 (0.0%) | 0 (0.0%) | 0 (0.0%) |
|  | A lot of energy (VT2) |  |  |  |
|  | All of the time | 2 (5.3%) | 3 (4.1%) | 5 (4.5%) |
|  | Most of the time | 10 (26.3%) | 16 (21.9%) | 26 (23.4%) |
|  | A good bit of the time | 11 (28.9%) | 14 (19.2%) | 25 (22.5%) |
|  | Some of the time | 12 (31.6%) | 20 (27.4%) | 32 (28.8%) |
|  | A little of the time | 3 (7.9%) | 17 (23.3%) | 20 (18.0%) |
|  | None of the time | 0 (0.0%) | 3 (4.1%) | 3 (2.7%) |
|  | Downhearted and blue (MH4) |  |  |  |
|  | All of the time | 0 (0.0%) | 0 (0.0%) | 0 (0.0%) |
|  | Most of the time | 3 (7.9%) | 9 (12.3%) | 12 (10.8%) |
|  | A good bit of the time | 9 (23.7%) | 14 (19.2%) | 23 (20.7%) |
|  | Some of the time | 9 (23.7%) | 24 (32.9%) | 33 (29.7%) |
|  | A little of the time | 15 (39.5%) | 20 (27.4%) | 35 (31.5%) |
|  | None of the time | 2 (5.3%) | 6 (8.2%) | 8 (7.2%) |
|  | Physical health or emotional problems interfering with social activities (SF2) |  |  |  |
|  | All of the time | 0 (0.0%) | 2 (2.7%) | 2 (1.8%) |
|  | Most of the time | 8 (21.1%) | 11 (15.1%) | 19 (17.1%) |
|  | Some of the time | 10 (26.3%) | 28 (38.4%) | 38 (34.2%) |
|  | A little of the time | 19 (50.0%) | 23 (31.5%) | 42 (37.8%) |
|  | None of the time | 1 (2.6%) | 9 (12.3%) | 10 (9.0%) |
| Severe | General Health (GH1) |  |  |  |
|  | Excellent | 5 (16.1%) | 1 (1.3%) | 6 (5.5%) |
|  | Very good | 5 (16.1%) | 17 (21.5%) | 22 (20.0%) |
|  | Good | 13 (41.9%) | 42 (53.2%) | 55 (50.0%) |
|  | Fair | 7 (22.6%) | 16 (20.3%) | 23 (20.9%) |
|  | Poor | 1 (3.2%) | 3 (3.8%) | 4 (3.6%) |
|  | Limited in Moderate Activities (PF02) |  |  |  |
|  | Yes, limited a lot | 1 (3.2%) | 10 (12.7%) | 11 (10.0%) |
|  | Yes, limited a little | 19 (61.3%) | 34 (43.0%) | 53 (48.2%) |
|  | No, not limited at all | 11 (35.5%) | 35 (44.3%) | 46 (41.8%) |
|  | Limited in Climbing Stairs (PF04) |  |  |  |
|  | Yes, limited a lot | 1 (3.2%) | 9 (11.4%) | 10 (9.1%) |
|  | Yes, limited a little | 18 (58.1%) | 39 (49.4%) | 57 (51.8%) |
|  | No, not limited at all | 12 (38.7%) | 31 (39.2%) | 43 (39.1%) |
|  | Accomplished less as a result of Physical Health (VRP2) |  |  |  |
|  | No, none of the time | 1 (3.2%) | 7 (8.9%) | 8 (7.3%) |
|  | Yes, a little of the time | 7 (22.6%) | 20 (25.3%) | 27 (24.5%) |
|  | Yes, some of the time | 16 (51.6%) | 31 (39.2%) | 47 (42.7%) |
|  | Yes, most of the time | 7 (22.6%) | 15 (19.0%) | 22 (20.0%) |
|  | Yes, all of the time | 0 (0.0%) | 6 (7.6%) | 6 (5.5%) |
|  | Limited in the kind of work or other activities as a result of Physical Health (VRP3) |  |  |  |
|  | No, none of the time | 2 (6.5%) | 11 (13.9%) | 13 (11.8%) |
|  | Yes, a little of the time | 6 (19.4%) | 20 (25.3%) | 26 (23.6%) |
|  | Yes, some of the time | 16 (51.6%) | 33 (41.8%) | 49 (44.5%) |
|  | Yes, most of the time | 7 (22.6%) | 14 (17.7%) | 21 (19.1%) |
|  | Yes, all of the time | 0 (0.0%) | 1 (1.3%) | 1 (0.9%) |
|  | Accomplished less as a result of any Emotional Problems (VRE2) |  |  |  |
|  | No, none of the time | 2 (6.5%) | 6 (7.6%) | 8 (7.3%) |
|  | Yes, a little of the time | 9 (29.0%) | 18 (22.8%) | 27 (24.5%) |
|  | Yes, some of the time | 12 (38.7%) | 34 (43.0%) | 46 (41.8%) |
|  | Yes, most of the time | 6 (19.4%) | 16 (20.3%) | 22 (20.0%) |
|  | Yes, all of the time | 2 (6.5%) | 5 (6.3%) | 7 (6.4%) |
|  | Limited in the kind of work or other activities as a result of any Emotional Problems (VRE3) |  |  |  |
|  | No, none of the time | 3 (9.7%) | 15 (19.0%) | 18 (16.4%) |
|  | Yes, a little of the time | 6 (19.4%) | 24 (30.4%) | 30 (27.3%) |
|  | Yes, some of the time | 13 (41.9%) | 27 (34.2%) | 40 (36.4%) |
|  | Yes, most of the time | 7 (22.6%) | 11 (13.9%) | 18 (16.4%) |
|  | Yes, all of the time | 2 (6.5%) | 2 (2.5%) | 4 (3.6%) |
|  | Pain interfering with normal work (BP2) |  |  |  |
|  | Not at all | 2 (6.5%) | 7 (8.9%) | 9 (8.2%) |
|  | A little bit | 8 (25.8%) | 21 (26.6%) | 29 (26.4%) |
|  | Moderately | 14 (45.2%) | 30 (38.0%) | 44 (40.0%) |
|  | Quite a bit | 6 (19.4%) | 15 (19.0%) | 21 (19.1%) |
|  | Extremely | 1 (3.2%) | 6 (7.6%) | 7 (6.4%) |
|  | Calm and peaceful (MH3) |  |  |  |
|  | All of the time | 6 (19.4%) | 1 (1.3%) | 7 (6.4%) |
|  | Most of the time | 6 (19.4%) | 10 (12.7%) | 16 (14.5%) |
|  | A good bit of the time | 6 (19.4%) | 13 (16.5%) | 19 (17.3%) |
|  | Some of the time | 6 (19.4%) | 29 (36.7%) | 35 (31.8%) |
|  | A little of the time | 4 (12.9%) | 21 (26.6%) | 25 (22.7%) |
|  | None of the time | 3 (9.7%) | 5 (6.3%) | 8 (7.3%) |
|  | A lot of energy (VT2) |  |  |  |
|  | All of the time | 0 (0.0%) | 2 (2.5%) | 2 (1.8%) |
|  | Most of the time | 10 (32.3%) | 7 (8.9%) | 17 (15.5%) |
|  | A good bit of the time | 7 (22.6%) | 14 (17.7%) | 21 (19.1%) |
|  | Some of the time | 6 (19.4%) | 25 (31.6%) | 31 (28.2%) |
|  | A little of the time | 6 (19.4%) | 22 (27.8%) | 28 (25.5%) |
|  | None of the time | 2 (6.5%) | 9 (11.4%) | 11 (10.0%) |
|  | Downhearted and blue (MH4) |  |  |  |
|  | All of the time | 5 (16.1%) | 6 (7.6%) | 11 (10.0%) |
|  | Most of the time | 5 (16.1%) | 12 (15.2%) | 17 (15.5%) |
|  | A good bit of the time | 8 (25.8%) | 15 (19.0%) | 23 (20.9%) |
|  | Some of the time | 7 (22.6%) | 27 (34.2%) | 34 (30.9%) |
|  | A little of the time | 6 (19.4%) | 19 (24.1%) | 25 (22.7%) |
|  | None of the time | 0 (0.0%) | 0 (0.0%) | 0 (0.0%) |
|  | Physical health or emotional problems interfering with social activities (SF2) |  |  |  |
|  | All of the time | 3 (9.7%) | 7 (8.9%) | 10 (9.1%) |
|  | Most of the time | 9 (29.0%) | 22 (27.8%) | 31 (28.2%) |
|  | Some of the time | 13 (41.9%) | 31 (39.2%) | 44 (40.0%) |
|  | A little of the time | 6 (19.4%) | 18 (22.8%) | 24 (21.8%) |
|  | None of the time | 0 (0.0%) | 1 (1.3%) | 1 (0.9%) |

**Legend**: Abbreviations: MIDAS = Migraine Disability Assessment; VR-12 = Veterans Rand 12-item Health Survey. Bracketed abbreviations are the shorthand reference names assigned by the VR-12 developers to each question.

**Supplementary Table 3**. Multiple regression models for the association between MIDAS disability level and health utility index score derived from the VR-12.

| **Independent variable** | **Overall Sample**  **Coefficient (95%CI)** | **Women**  **Coefficient (95%CI)** | **Men**  **Coefficient (95%CI)** |
| --- | --- | --- | --- |
| **Intercept** | 0.73 (0.64, 0.83)*** | 0.62 [0.49,0.74]*** | 0.84 [0.69,0.99]*** |
| **Migraine disability level** |  |  |  |
| Little to no | [Reference] | [Reference] | [Reference] |
| Mild | -0.08 [-0.14,-0.03]** | 0.01 [-0.07,0.09] | -0.16 [-0.24,-0.09]*** |
| Moderate | -0.11 [-0.16,-0.05]*** | -0.03 [-0.10,0.05] | -0.18 [-0.26,-0.10]*** |
| Severe | -0.23 [-0.28,-0.17]*** | -0.16 [-0.24,-0.09]*** | -0.28 [-0.37,-0.20]*** |
| **Gender** |  |  |  |
| Man | [Reference] | NA | NA |
| Woman | -0.03 [-0.06,0.01] |  |  |
| **Age, per year** | -0.0011 [-0.0034,0.0011] | -0.0005 [-0.0034,0.0024] | -0.0022 [-0.0060,0.0017] |
| **Ethnicity** |  |  |  |
| Other race/ ethnicity* | [Reference] | [Reference] | [Reference] |
| White | 0.02 [-0.03,0.06] | -0.01 [-0.07,0.05] | 0.06 [-0.01,0.13] |
| **Education** |  |  |  |
| No university/college education | [Reference] | [Reference] | [Reference] |
| University/ college education | 0.05 [0.01, 0.09]* | 0.06 [0.01,0.12]* | 0.02 [-0.04,0.08] |
| **Marital status** |  |  |  |
| Not married or common-law | [Reference] | [Reference] | [Reference] |
| Married or common-law | -0.01[-0.05,0.03] | -0.02 [-0.07,0.04] | 0.03 [-0.04,0.10] |
| **Household income** |  |  |  |
| <$50,000 | [Reference] | [Reference] | [Reference] |
| $50,000- $99,999 | 0.05 [-0.01,0.11] | 0.08 [0.01,0.15]* | -0.03 [-0.13,0.07] |
| $100,000- $149,999 | 0.06 [-0.00,0.12] | 0.09 [0.02,0.17]* | -0.03 [-0.14,0.07] |
| ≥$150,000 | 0.07 [0.01,0.14]* | 0.09 [0.01,0.18]* | -0.01 [-0.11,0.09] |
| **Migraine duration, per year** | 0.0015 [-0.0005,0.0036] | 0.0023 [-0.0003,0.0048] | 0.0006 [-0.0031,0.0044] |
| **Number of comorbidities** |  |  |  |
| 0 | [Reference] | [Reference] | [Reference] |
| 1 | -0.06 [-0.10,-0.02]** | -0.10 [-0.16,-0.04]** | -0.02 [-0.08,0.04] |
| ≥2 | -0.13 [-0.17,-0.08]*** | -0.14 [-0.20,-0.08]*** | -0.11 [-0.19,-0.03]** |
|  |  |  |  |

**Legend**: Abbreviations: MIDAS = Migraine Disability Assessment; VR-12 = Veterans Rand 12-item Health Survey. Models were adjusted for age, White ethnicity, education attainment, marital status, household income, migraine duration (since diagnosis), and the number of comorbidities. Models including the overall sample also adjusted for gender. **p*<0.05; ***p*<0.01; ****p*<0.001.

**Supplementary Table 4**. Multiple regression models for the association between MIDAS headache pain and health utility index score derived from the VR-12.

| **Independent variable** | **Overall Sample**  **Coefficient (95%CI)** | | **Women**  **Coefficient (95%CI)** | | **Men**  **Coefficient (95%CI)** |
| --- | --- | --- | --- | --- | --- |
| **Intercept** | 0.77 [0.66,0.87]*** | | 0.66 [0.52,0.81]*** | | 0.86 [0.69,1.03]*** |
| **Headache pain** | -0.03 [-0.04,-0.02]*** | | -0.02 [-0.03,-0.01]** | | -0.03 [-0.05,-0.02]*** |
| **Gender** |  | |  | |  |
| Man | [Reference] | | NA | | NA |
| Woman | -0.02 [-0.06,0.02] | |  | |  |
| **Age, per year** | -0.0003 [-0.0026,0.0021] | | 0.0001 [-0.0028,0.0031] | | -0.0002 [-0.0043,0.0038] |
| **Ethnicity** |  | |  | |  |
| Other race/ ethnicity* | [Reference] | | [Reference] | | [Reference] |
| White | 0.00 [-0.05,0.05] | | -0.01 [-0.07,0.06] | | 0.02 [-0.06,0.10] |
| **Education** |  | |  | |  |
| No university/college education | [Reference] | | [Reference] | | [Reference] |
| University/ college education | 0.03 [-0.01,0.07] | | 0.05 [-0.01,0.10] | | 0.00 [-0.06,0.06] |
| **Marital status** |  | |  | |  |
| Not married or common-law | [Reference] | | [Reference] | | [Reference] |
| Married or common-law |  | |  | |  |
| **Household income** |  | |  | |  |
| <$50,000 | [Reference] | | [Reference] | | [Reference] |
| $50,000- $99,999 | 0.05 [-0.01,0.11] | | 0.06 [-0.01,0.13] | | 0.01 [-0.10,0.11] |
| $100,000- $149,999 | 0.05 [-0.01,0.11] | | 0.08 [0.00,0.16]* | | -0.04 [-0.15,0.07] |
| ≥$150,000 | 0.07 [0.00,0.13]* | | 0.08 [-0.01,0.17] | | 0.00 [-0.11,0.11] |
| **Migraine duration, per year** | 0.0021 [-0.0001,0.0042] | | 0.0026 [-0.0000,0.0053] | | -0.0000 [-0.0040,0.0039] |
| **Number of comorbidities** |  | |  | |  |
| 0 | [Reference] | | [Reference] | | [Reference] |
| 1 | -0.05 [-0.10,-0.01]* | | -0.08 [-0.15,-0.02]** | | -0.02 [-0.09,0.04] |
| ≥2 | -0.14 [-0.19,-0.09]*** | | -0.15 [-0.21,-0.09]*** | | -0.12 [-0.21,-0.03]* |
|  |  |  | |  | |

**Legend**: Abbreviations: MIDAS = Migraine Disability Assessment; VR-12 = Veterans Rand 12-item Health Survey. Models were adjusted for age, White ethnicity, education attainment, marital status, household income, migraine duration (since diagnosis), and the number of comorbidities. Models including the overall sample also adjusted for gender. **p*<0.05; ***p*<0.01; ****p*<0.001.

**Supplementary Table 5**. Multiple regression models for the association between MIDAS headache frequency and health utility index score derived from the VR-12.

| **Independent variable** | **Overall Sample**  **Coefficient (95%CI)** | | **Women**  **Coefficient (95%CI)** | | **Men**  **Coefficient (95%CI)** |
| --- | --- | --- | --- | --- | --- |
| **Intercept** | 0.66 [0.57,0.75]*** | | 0.58 [0.47,0.69]*** | | 0.75 [0.59,0.91]*** |
| **Headache frequency** | -0.0052 [-0.0067,-0.0037]*** | | -0.0048 [-0.0065,-0.0030]*** | | -0.0063 [-0.0094,-0.0032]*** |
| **Gender** |  | |  | |  |
| Man | [Reference] | | NA | | NA |
| Woman | -0.04 [-0.08,0.00] | |  | |  |
| **Age, per year** | -0.0000 [-0.0023,0.0022] | | 0.0006 [-0.0023,0.0035] | | -0.0010 [-0.0051,0.0031] |
| **Ethnicity** |  | |  | |  |
| Other race/ ethnicity* | [Reference] | | [Reference] | | [Reference] |
| White | 0.02 [-0.02,0.07] | | 0.01 [-0.05,0.06] | | 0.07 [-0.01,0.14] |
| **Education** |  | |  | |  |
| No university/college education | [Reference] | | [Reference] | | [Reference] |
| University/ college education | 0.03 [-0.01,0.07] | | 0.04 [-0.01,0.10] | | 0.01 [-0.05,0.08] |
| **Marital status** |  | |  | |  |
| Not married or common-law | [Reference] | | [Reference] | | [Reference] |
| Married or common-law | -0.04 [-0.08,0.00] | | -0.03 [-0.08,0.03] | | -0.03 [-0.10,0.04] |
| **Household income** |  | |  | |  |
| <$50,000 | [Reference] | | [Reference] | | [Reference] |
| $50,000- $99,999 | 0.05 [-0.01,0.11] | | 0.07 [-0.00,0.14] | | -0.02 [-0.13,0.09] |
| $100,000- $149,999 | 0.07 [0.01,0.13]* | | 0.10 [0.03,0.18]** | | -0.02 [-0.13,0.09] |
| ≥$150,000 | 0.10 [0.03,0.16]** | | 0.11 [0.02,0.19]* | | 0.01 [-0.10,0.12] |
| **Migraine duration, per year** | 0.0008 [-0.0013,0.0029] | | 0.0014 [-0.0012,0.0039] | | -0.0007 [-0.0048,0.0033] |
| **Number of comorbidities** |  | |  | |  |
| 0 | [Reference] | | [Reference] | | [Reference] |
| 1 | -0.05 [-0.10,-0.01]* | | -0.08 [-0.14,-0.02]* | | -0.02 [-0.09,0.04] |
| ≥2 | -0.13 [-0.18,-0.08]*** | | -0.13 [-0.19,-0.08]*** | | -0.12 [-0.21,-0.03]** |
|  |  |  | |  | |

**Legend**: Abbreviations: MIDAS = Migraine Disability Assessment; VR-12 = Veterans Rand 12-item Health Survey. Models were adjusted for age, White ethnicity, education attainment, marital status, household income, migraine duration (since diagnosis), and the number of comorbidities. Models including the overall sample also adjusted for gender. **p*<0.05; ***p*<0.01; ****p*<0.001.

**Supplementary Table 6**. Multiple regression model for the association between MIDAS disability level and health utility index score derived from the VR-12 with interaction terms for gender and MIDAS.

| **Independent variable** | **Overall Sample**  **Coefficient (95%CI)** |
| --- | --- |
| **Intercept** | 0.50 [0.39, 0.60]*** |
| **Migraine disability level** |  |
| Little to no | 0.28 [0.19,0.36]*** |
| Mild | 0.11 [0.02,0.20]* |
| Moderate | 0.11 [0.01,0.20]* |
| Severe | [Reference] |
| **Gender** |  |
| Men | [Reference] |
| Women | -0.02 [-0.10,0.06] |
| **Interaction terms** |  |
| Little to no # Women | -0.11 [-0.22, 0.00] |
| Mild # Women | 0.05 [-0.06, 0.16] |
| Moderate # Women | 0.02 [-0.09, 0.13] |
| Severe # Women | [Reference] |
|  |  |

**Legend**: Abbreviations: MIDAS = Migraine Disability Assessment; VR-12 = Veterans Rand 12-item Health Survey. Models were adjusted for age, gender, White ethnicity, education attainment, marital status, household income, migraine duration (since diagnosis), the number of comorbidities, and interactions term between MIDAS and gender. **p*<0.05; ***p*<0.01; *** *p*<0.001.

**Supplementary Table 7**. Multiple regression model for the association between different MIDAS measures and health utility index score derived from the VR-12 with interaction terms for gender and MIDAS.

| **Independent variable** | **MIDAS score**  **Coefficient (95%CI)** | **Headache pain**  **Coefficient (95%CI)** | | **Headache frequency**  **Coefficient (95%CI)** | |
| --- | --- | --- | --- | --- | --- |
| **Intercept** | 0.6902 [0.5995,0.7808] *** | 0.7906 [0.6803,0.9008] *** | | 0.6647 [0.5732,0.7561] *** | |
| **MIDAS disability score** | -0.0040 [-0.0057,-0.0024] *** | - | | - | |
| **Headache pain** | - | -0.0319 [-0.0449,-0.0189] *** | | - | |
| **Headache frequency** | - | - | | -0.0062 [-0.0092,-0.0032] *** | |
| **Gender** |  |  | |  | |
| Men | [Reference] | [Reference] | | [Reference] | |
| Women | -0.0620 [-0.1095,-0.0145] * | -0.0808 [-0.1840,0.0223] | | -0.0474 [-0.0951,0.0002] | |
| **Interaction term # women** | 0.0017 [-0.0001,0.0035] | 0.0103 [-0.0065,0.0270] | | 0.0012 [-0.0022,0.0047] | |
|  |  | |  | |  |

**Legend**: Abbreviations: MIDAS = Migraine Disability Assessment; VR-12 = Veterans Rand 12-item Health Survey. Models were adjusted for age, gender, White ethnicity, education attainment, marital status, household income, migraine duration (since diagnosis), the number of comorbidities, and an interaction term between gender and MIDAS disability score, headache pain, and headache frequency. **p*<0.05; ***p*<0.01; ****p*<0.001.
